# Researchers-in-Residence to facilitate co-production: the TRUUD project

**DOI:** 10.1177/17579139221103183

**Published:** 2022-07-14

**Authors:** A Le Gouais, S Peake-Jones

**Affiliations:** Senior Research Associate, Population Health Sciences, Bristol Medical School, University of Bristol, 1–5 Whiteladies Road, Bristol BS8 1NU, UK; Research Fellow, Planning and Environment Management, The University of Manchester, Manchester, UK

## Urban Environment Decision-Making and Health Outcomes

We know that the built environment impacts on population health and wellbeing, particularly non-communicable diseases.^1^ For example, having safe walking or cycling routes will affect levels of physical activity and associated health outcomes,^2,3^ while access to urban greenspace is associated with physical and mental health.^4^

The burden of disease and financial costs associated with unhealthy urban environments is significant, for example, poor air quality alone is estimated to cost the UK over £20 billion annually.^5^ Despite the negative health impacts of certain environments, urban development decision-makers tend to come from non-health sectors and have non-health priorities when it comes to areas such as transport, urban planning or property development.

The system of urban development decision-making is complex, involving many stakeholders with competing priorities and influences. The TRUUD (‘Tackling Root causes Upstream of Unhealthy Urban Development’) project was established to try to untangle those influences that can result in unhealthy place-making and find ways to influence healthy decision-making. It is a 5-year research project (October 2019 to September 2024) involving around 40 researchers with diverse expertise including public health, transport, urban development, economics, policy studies, public involvement and systems engineering.

## Truud’s Researcher-In-Residence Model

A core facet of the TRUUD research is co-production of interventions with the public and our practice partners. A cornerstone to this is the inclusion of two Researchers-in-Residence (RIRs) embedded in partner local authorities: Anna Le Gouais is seconded into Bristol City Council part time and Sian Peake-Jones works with Greater Manchester Combined Authority. These roles started in 2020 and will continue until 2024.

**Figure:**
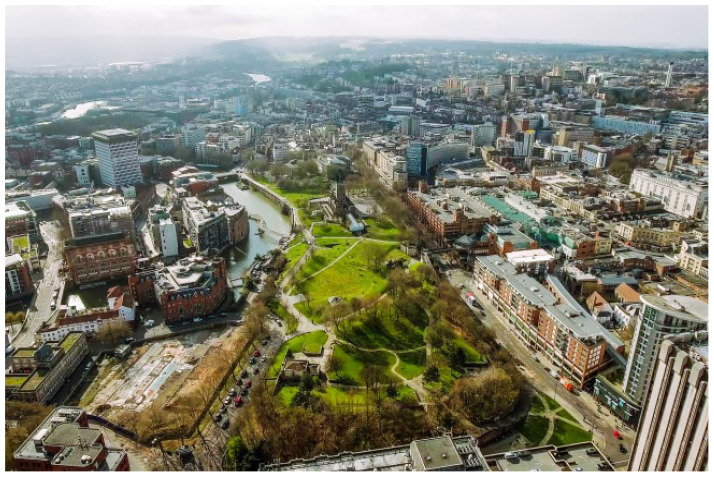


Embedded research is a methodological approach that includes observing, influencing and participating through being a ‘sounding board, knowledge broker, facilitator, capacity builder and catalyst for change and improvement’.^6^ It has been shown to facilitate timely and relevant research^7^ and is increasingly being used within local authority public health teams to support service improvement. However, to understand the complex system of urban development decision-making, the TRUUD RIRs sit outside of public health teams, instead working directly within urban development and transport policy teams.

## Building Partnerships to Understand a Complex System

Building trust is an important element of embedded research which takes time. This is crucial to gain exposure to key events and people that influence decision-making for urban development and facilitate understanding of organisational culture, norms and political realities,^6^ all of which affect decision-making for policy and practice. The time taken to develop trust is particularly important because of the confidentiality issues involved in sharing information between organisations and the potential political, reputational and commercial risks associated with this research. One way we have addressed this is to co-produce working protocols with our local authority partners, such as data management agreements around consent that are both reflexive and informed,^8^ ensuring the role of the researcher is overt and communicated clearly.

Forming relationships with colleagues and becoming accepted within our local government organisations have been slower due to the COVID-19 pandemic – ideally, we would have been sitting with local government colleagues at least a couple of days a week, but instead the majority of interactions have been online as people have predominantly worked from home (although not exclusively). Despite these challenges, we have both become embedded in our partner organisations with strong trusted working relationships, although forging connections between local government colleagues and the wider multi-disciplinary academic team is ongoing.

## Co-Producing Pragmatic Interventions

RIRs act as a bridge between research and practice, to ensure that pragmatic research is conducted that is relevant and useful for the partner organisation. As RIRs we are therefore not simply observers or knowledge brokers but are seeking to gain and use knowledge and relationships to facilitate co-produced interventions or service improvements that will have demonstrable impact in practice for healthier environments.

The RIR role supports the co-production of TRUUD interventions through day-to-day engagement with actors involved in decision-making for urban development, based on the principle that those who will be delivering a service or intervention are best placed to help design it. Through close working relationships, we can ensure that interventions are relevant, deliverable and impactful.^9^ This may involve elements of serendipity, where opportunities arise to influence policy and practice that were not identified a priori, and may take advantage of ‘windows of opportunity’.^10^

The co-production of TRUUD interventions is ongoing as the project seeks to influence multiple leverage points across the complex urban development system. An early example of facilitating co-production with local government partners is development of health economic models to demonstrate the economic impact of features of the built environment associated with health and wellbeing outcomes. As RIRs we are connecting our local government and transport authority colleagues with TRUUD researchers to work together to develop practical tools to influence healthy urban development decision-making. This is being done separately in Bristol and Greater Manchester, for property development and transport, respectively. We will be able to support the use of these tools in every day practice and use our embedded roles to evaluate how influential they could be to support healthy urban development by learning from our local government colleagues, with opportunity for iterative feedback and improvement.

## The Multiple Roles of Rirs

**Figure 1:**
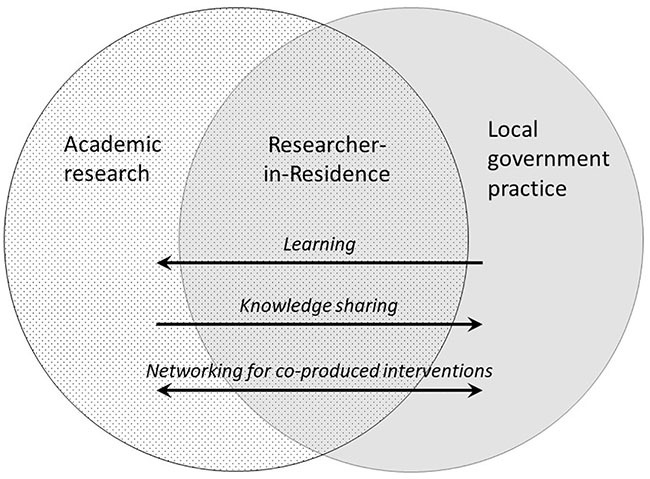
The multiple roles of a Researcher-in-Residence

The RIR role includes multiple dimensions (Figure 1). In our local authority roles, we may act as a researcher, observing situations to learn about urban development processes and practices to inform the wider TRUUD research project; as a knowledge broker, sharing research findings with practitioners across disciplines; as a networker or facilitator, connecting local government colleagues with TRUUD researchers to discuss potential opportunities for co-produced interventions; or simply as a colleague, working together to implement and evaluate projects. This is all alongside our researcher roles as part of the wider TRUUD project team, where we can act as a constant reminder to our academic colleagues of the need for pragmatic, relevant and impactful research.

## Summary

We have described the approach of TRUUD’s RIRs, working with non-health colleagues in two local government organisations in England. This embedded research model includes observing, influencing and participating to support co-production of interventions that target urban environment decision-making for healthier place-making. This has been facilitated by building trusted relationships with partners to understand a complex system. Through primary data collection, knowledge brokerage, networking and facilitation, RIRs can help large project teams to develop pragmatic co-produced interventions for impactful research.
